# Factors influencing the implementation of a lifestyle counseling program in patients with venous leg ulcers: a multiple case study

**DOI:** 10.1186/1748-5908-7-104

**Published:** 2012-10-26

**Authors:** Irene M van de Glind, Maud M Heinen, Andrea W Evers, Michel Wensing, Theo van Achterberg

**Affiliations:** 1 Scientific Institute for Quality of Healthcare, Radboud University Nijmegen Medical Centre, Nijmegen, the Netherlands; 2 Department of Medical Psychology, Radboud University Nijmegen Medical Centre, Nijmegen, the Netherlands

**Keywords:** Lifestyle, Varicose ulcer, Venous leg ulcers, Implementation, Case study

## Abstract

**Background:**

Implementation of lifestyle interventions in patient care is a major challenge. Understanding factors that influence implementation is a first step in programs to enhance uptake of these interventions. A lifestyle-counseling intervention, Lively Legs, delivered by trained nurses, can effectively improve the lifestyle in patients with venous leg ulcers. The aim of this study was to identify factors that hindered or facilitated implementation of this intervention in outpatient dermatology clinics and in home care.

**Methods:**

A mixed-methods multiple case study in five purposefully selected healthcare settings in the Netherlands was conducted. Measurements to identify influencing factors before and after implementation of Lively Legs included interviews, focus groups, questionnaires, and nurses’ registration. Analyses focused on qualitative data as the main data source. All data were compared across multiple cases to draw conclusions from the study as a whole.

**Results:**

A total of 53 patients enrolled in the Lively Legs program, which was delivered by 12 trained nurses. Barriers for implementation were mainly organizational. It was difficult to effectively organize reaching and recruiting patients for the program, especially in home care. Main barriers were a lack of a standardized healthcare delivery process, insufficient nursing time, and a lack of motivated nurses to deliver the program. Facilitating factors were nurse-driven coordination of care and a standardized care process to tie Lively Legs into, as this resulted in better patient recruitment and better program implementation.

**Conclusions:**

This study identified a range of factors influencing the implementation of a lifestyle-counseling program, mainly related to the organization of healthcare. Using a case study method proved valuable in obtaining insight into influencing factors for implementation. This study also shed light on a more general issue, which is that leg ulcer care is often fragmented, indicating that quality improvement is needed.

## Background

Physical exercise and compliance with compression therapy are key elements in the course of leg ulcer healing and recurrence. However, many patients demonstrate sedentary lifestyles and noncompliance [1-7]. Although patients report several problems that are related to lifestyle, studies show that the current care practice for this patient group does not address these topics sufficiently well [8-10]. In order to improve the adherence of patients with venous leg ulcers to a healthy lifestyle, the nurse-led counseling program Lively Legs was developed for outpatient dermatology clinics [11].

The Lively Legs program primarily aims at increasing physical activity (particularly walking), leg exercises, and adherence with compression therapy in venous leg ulcer patients. Next to this, the program focuses on adequate pain management, proper hygiene of feet and legs, adequate footwear, and a healthy diet [11]. The program was systematically developed in collaboration with several representatives (*i*.*e*., patients, dermatologists, nurses, a clinical psychologist, a physiotherapist, a dietician, hospital management, nursing associations, and patient organizations) [11] and is based on the Social Cognitive Theory, Goal Setting Theory, and the Precaution Adoption Process Model [12-17].

The effectiveness of the Lively Legs program on behavior change was established in a multicenter randomized controlled trial [18]. Results of this study showed that the intervention group performed better on conducting leg exercises and 10-minute walks five days a week. Adherence with compression therapy increased in both the intervention group and the control group, but there was no difference between the groups. There were fewer wound days in the intervention group after the initial wound had healed [18]. Studies in other countries present similar results. For example, Van Hecke *et al*. (2011) reported an increase in the number of patients that performed exercises after a nurse-led intervention comparable to Lively Legs [19]. The Leg Club model, developed in the United Kingdom [20], is a community care model that makes use of peer support, assistance with goal setting, and social interaction. This model was adopted and evaluated in community nursing in Queensland in Australia [21]. Positive significant effects were reported on quality of life, functional ability, and on morale and self-esteem. Effects on ulcer healing and pain were not conclusive, but were promising. Finally, in Germany, researchers recently developed a nurse-led educational intervention to enhance self-care in patients with chronic ulcers [22]. Thus, similar initiatives in other countries have been reported [19-24], underlining the need for health promotion in this population. However, implementation of such preventive interventions in routine healthcare is a major challenge [25-28].

A systematic and stepwise approach is recommended to effectively implement innovations in healthcare [29]. One of the first steps is to perform a diagnostic analysis to identify factors that facilitate or hinder implementation. Often, this is done before implementation using questionnaires, qualitative interviews, or focus groups. However, little is known of the extent to which these identified influencing factors are really important. Therefore, it is interesting to investigate factors before, but also during and after, implementation. This leads to an in-depth picture of how and why implementation is feasible in different settings, which contributes to better implementation [30,31].

**Figure 1:**
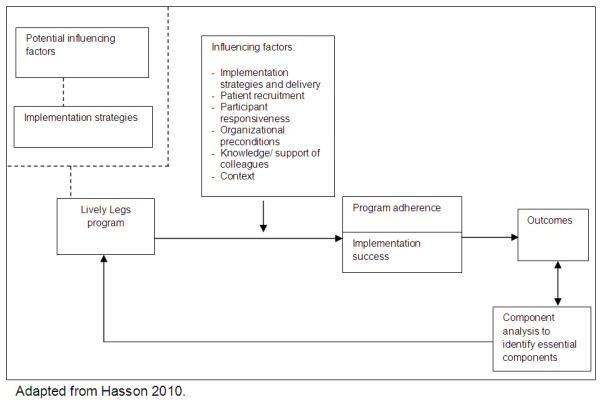
**Conceptual framework for evaluation of implementation.** A framework to evaluate the implementation of a lifestyle program. This framework, adapted from Hasson 2010, describes different areas that could influence the outcome of a lifestyle program: Influencing factors (*e*.*g*. Implementation strategies and delivery, Patient recruitment, Participant responsiveness, Organizational preconditions, Knowledge/support of colleagues, Context), Program adherence and Implementation success.

Multiple components need to be assessed in order to achieve a comprehensive picture of an implementation process in a real-life setting [26,32-35]. Hasson (2010) proposed a framework for evaluation of implementation, which is based on the framework of Carroll *et al*. (2007) (see Figure 1) [33,36].

In the adapted framework, the first step is to systematically explore potential factors that facilitate or hinder program implementation [37]. This phase is followed by selecting implementation strategies that are connected to these factors [37,38]. Outcomes of an implementation process can be divided in program adherence, implementation success, and in the end patient outcomes. Implementation success is closely related to program adherence but emphasizes how well the program is embedded in the organization. This distinction is made because organizational implementation can be judged as insufficient, even though program adherence is adequate. Outcomes may be affected by several influencing factors: participant responsiveness (how satisfied were the participants), comprehensiveness of policy description (how specific is the intervention description), strategies to facilitate implementation, the quality of delivery of implementation strategies, patient recruitment, and context (what factors at political, economic, organizational, and work-group levels affected the implementation).

Using Hasson’s framework, we prepared and evaluated the implementation of the Lively Legs program for lifestyle counseling in venous leg ulcer patients. This research contributes to the body of knowledge on implementing preventive counseling programs in healthcare. The aim of this study was to identify which factors hindered and facilitated implementation of Lively Legs in outpatient clinics for dermatology and in homecare.

## Methods

This study used a multiple case study to obtain a picture of how and why implementation is successful in different settings [39]. A case study was defined as “an empirical inquiry that investigates a contemporary phenomenon in depth and within its real-life context” ([39] p. 18). This design was chosen because implementing a lifestyle program in a healthcare setting could be regarded as an event over which researchers can have little control. Next to this, an implementation process could be considered to be a situation in which there will be many more variables of interest than data points, particularly because a lifestyle intervention is a complex intervention [32], and boundaries between the implementation process, the lifestyle intervention, and context are not always clearly evident. The value of a case study design lies in exploring and describing the relevant variables of interest and “telling the story” of what happened.

In five healthcare settings (five cases) in the Netherlands, the implementation of the Lively Legs program was examined (three cases in 2009 and an additional two cases in 2010). A case was defined as a region around an outpatient clinic for dermatology where the program was implemented. Starting from the outpatient clinic for dermatology, possibilities for collaboration with homecare agencies were explored in each case. Thus, sometimes one case consists of more than one healthcare setting because of collaboration with one or more homecare agencies. Each case was studied using both qualitative and quantitative methods; however, qualitative data (interviews, focus groups, field notes, and observations) were the main data source to identify influencing factors for implementation (See Table 1 for an overview of the data collection). In the first year, evaluation was conducted seven months after implementation (cases 1, 2, and 3); in the second year, this was after four months (cases 4 and 5). The Medical Ethics Committee of district Arnhem-Nijmegen assessed the study and concluded that our study was deemed exempt from their approval.

**Table 1:** Characteristics data collection

	**Case 1**	**Case 2**	**Case 3**	**Case 4**	**Case 5**	**Total**
**Identifying influencing factors beforehand**						
Focus groups	1	1	-	1	-	3
Semistructured interviews with nurses, dermatologists, managers, referrers	4	8	5	8	7	32
Facilitators and barriers questionnaire in nurses participating in the previous trial						10 (65%)
Semistructured interviews with healthcare insurers						6
**Evaluation**						
Monitor of patient recruitment (online tool) filled in by nurses	Yes	Yes	No	Yes	Yes	
Semistructured interviews and semistructured group interviews with nurses, dermatologists, managers, referrers	7	7	2^a^	4	2^a^	22
Number of patients enrolled in the program	10	18	14	6	5	53
Number of patients who completed the program within evaluation	10	18	7	5	5	45
Evaluation forms filled in by nurses	10	18	5	5	5	43
Patient satisfaction questionnaires	6	9	3	2	0	20
Facilitators and barriers questionnaire— nurses	2	4	1	2	3	12 (86%)^b^
Facilitators and barriers questionnaire— colleagues	5	8	3	3	3	22 (61%)
Observation of counseling sessions	1	1	1	1	2	6

^a^One was a group interview with nurse(s), manager, and/or dermatologist. ^b^Despite efforts, no questionnaire was filled in by one nurse in case 2 and one nurse in case 4.

### Description of the Lively Legs intervention

The Lively Legs program offers a method to promote healthy behaviors in patients with leg ulcers of a predominantly venous etiology. Patients receive two to six individual counseling sessions from a trained nurse in addition to usual care. The program starts with a lifestyle assessment (45–60 minutes) to determine patients’ current health behaviors, among other things, the level of physical activity and adherence to compression therapy. During this assessment, patients receive tailored health education, including practicing how to perform leg exercises. Also, written educational materials are given to the patient at the end of the first session. In follow-up consultations (20–30 minutes), barriers and facilitators for potential behavior change are discussed using motivational interviewing, and one or two topics for behavior change are chosen to be worked on. The frequency of sessions and choice of topics is decided upon by nurse and patient. Goal setting and home assignments are part of the intervention, as well as discussing patients’ motivation and self-efficacy towards behavior change. The individual counseling consultations take place at the outpatient clinic or at patients’ homes.

The essential elements of the Lively Legs program are listed below.

– Session 1

 – Assessment of patients’ lifestyles

 – Give tailored health education related to patients’ health beliefs

 – Demonstrate and practice leg exercises

 – Give education materials to the patient

 – Discuss motivation and self-efficacy towards behavior change

 – Explore barriers and facilitators for behavior change

 – Goal setting on one or more lifestyle topics and home assignments

– Session 2–5

 – Evaluate behavior change and give feedback

 – Motivational interviewing

 – Discuss motivation and self-efficacy towards behavior change

 – Explore barriers and facilitators for behavior change

 – Goal setting on one or more lifestyle topics and home assignments

– Final (Preferably six months after session 1)

 – Evaluate behavior change and give feedback

 – Summarize the course of the counseling trajectory and the achieved results

 – Discuss maintenance of behavior change

### Selection of the cases

Five cases were purposefully selected for this study. Cases were selected to compose a heterogeneous sample in terms of geography, familiarity with the Lively Legs program, and degree of cooperation between dermatology outpatient clinic and homecare. This cooperation ranged from no cooperation at all to a joint standardized care process. An overview of the cases and the participants involved in the study is presented in the Results section (Table 2).

**Table 2:** Description of the cases and respondents

**Case**	**Healthcare settings**	**Familiar with program**?^**a**^	**Responsible for program delivery**	**Individuals involved in implementation**
**1**	1. Homecare setting	No	Nurse practitioner	Nurse practitioner, manager, GP
	2. Homecare setting	No	Homecare nurse	Homecare nurse, manager
	*Outpatient clinic* (*only to refer patients*)	*Yes*	-	*Dermatologist*, *three nurses*, *team manager*
**2**	3. Outpatient clinic	Yes	Dermatology nurse	Dermatologist, two nurses, team manager
	4. Outpatient clinic	No	Dermatology nurse	Dermatologist, two nurses, team manager (the same as in case 4)
	5. Homecare setting	No	Homecare nurse	Homecare nurse, manager, policy advisor
	6. Homecare setting	No	Homecare nurse	Homecare nurse
	7. Specialized primary wound care clinic	No	Specialized nurse	Specialized nurse, clinic manager, specialist elderly care
**3**	8. Outpatient clinic	Yes	Dermatology nurse	Dermatologist, dermatology nurse, team manager, hospital manager, homecare manager
**4**	9. Outpatient clinic	No	Dermatology nurse	Dermatologist, dermatology nurse, team manager
	10. Homecare setting	No	Homecare nurse	Homecare nurse, team manager, manager
	11. Homecare setting	No	Homecare nurse	Homecare nurse, manager
**5**	12. Outpatient clinic	No	Nurse practitioner	Dermatologist, nurse practitioner, medical assistants, team manager
			Two medical assistants	

^a^ Participated in the previous trial on Lively Legs.

### Identifying potential influencing factors for change

Potential influencing factors were identified by organizing focus group discussions and semistructured interviews with healthcare providers and managers in each of the cases. Focus groups and interviews mainly addressed two elements: identifying influencing factors and (group-based) brainstorming to identify solutions or strategies to the factors. Participants and respondents included a purposeful sample of dermatologists, dermatology nurses, homecare nurses, and managers. Additionally, all nurses who had participated in the previous effectiveness study were asked to fill in a questionnaire to classify influencing factors for implementation, as they already worked with the program and were expected to estimate potential factors based on their experience. The questionnaire was developed by the Scientific Institute for Quality of Healthcare (Nijmegen, The Netherlands) and was used in a range of studies on influencing factors for implementing innovation in healthcare [40,41]. A third group that was included to identify influencing factors for implementation were representatives of health insurers. They were interviewed to explore regulatory and financial factors that influence implementation.

### Developing implementation strategies

All identified influencing factors and possible implementation strategies were listed by the research team. To select implementation strategies, we used two existing classifications for change strategies in the literature to broaden the scope of strategies and thus reduce the chance of overlooking important issues [42,43]. The identified factors and possible implementation strategies were linked by two researchers (IMV, MMH). Relevant theories were considered for the selection of strategies to facilitate implementation [38]. The selected strategies were then presented in a timetable to clarify who should do what at what time and whether strategies were compulsory or to be used as desired. A first draft of the proposed implementation plan was sent to key persons in each of the cases asking for feedback. At the end, a final version of the implementation plan was sent to these key persons, and they were explicitly asked to approve of the plan and to contact the researchers when deviations would occur.

### Evaluation of the implementation of Lively Legs

Qualitative data (primarily interviews and secondary field notes and observations) were the main data source to identify influencing factors postimplementation. Nurses, dermatologists, and managers were interviewed (IMV) to explore factors that influenced implementation. Open-ended questions that were asked during these interviews were as follows: Can you tell me about your experiences with the program? What do you think of the program? What has driven/facilitated the implementation of Lively Legs? What were/are barriers? Why is that? Are those barriers resolved, and how? What was/is the most important barrier? What is needed to resolve this? What would you do differently next time? What would you advise to others that would want to implement this program? Besides the interviews, one researcher (IMV) observed counseling sessions to get an impression of how the program was delivered.

Next to this, the evaluation makes use of more structured and quantitative methods. Following the model of Hasson (2010), the following categories of potential influencing factors were assessed:

– Quality of the delivery of implementation strategies (implementation fidelity): Strategy delivery was assessed through registration by the researchers and checking to what extent strategies were carried out as planned in interviews with nurses and managers.

– Participant responsiveness: Responsiveness is the extent to which the Lively Legs program was appealing to patients and nurses. To measure this, patients were given a self-developed evaluation questionnaire with a return envelope at the end of the last counseling session. In the questionnaire, patients had to score how they valued the program on a scale from 1 to 10. Nurses also filled in an evaluation form to assess their satisfaction with the results achieved per patient on a scale from 1 to 5. Furthermore, the facilitators and barriers questionnaire also contains one question about how nurses valued the program in general on a scale from 1 to 5.

– Organizational preconditions: In interviews with nurses and managers, it was asked if there was sufficient nursing time to deliver the program and if a consulting room was available. In the facilitators and barriers questionnaire, there was one question on how nurses valued the extent to which the organization met the preconditions for delivering the program (scale from 1 to 5).

– Knowledge and support of colleagues: All participating nurses were asked to hand out the facilitators and barriers questionnaire with a return envelope to three colleagues at the end of the project. This questionnaire also contained one question about how well they knew what the program Lively Legs was about (colleagues) and one question about how supportive colleagues were in program delivery (nurses).

In addition to our main focus on identifying influencing factors, we examined whether the implementation was perceived as successful by nurses and how many patients were enrolled in the program.

– Perceived implementation success: Nurses were asked to give a score on a scale from 1 to 10 for how well the program was currently implemented. In addition, they were asked to give a score on a scale from 1 to 10 for how well the program will be implemented in a half a year’s time, reflecting their expectation of the embedding of the program.

– Patient recruitment: Patient recruitment was monitored weekly by nurses who filled in the number of eligible patients and the number of recruited and participating patients using an online tool. Because on average one new patient per month participated in the previous effectiveness trial [18], this was chosen as minimum standard for this study.

Data collection and measures on program adherence are described in more detail in Additional file 1. A coverage score was computed, which is the percentage of the components that are delivered as planned. For this study, a coverage score of 80% to 100% was regarded as high program adherence, a coverage score between 50% and 80% was regarded as moderate, and 50% or less as low program adherence.

### Data analyses

With respect to identifying influencing factors from qualitative data, the interviews, focus groups, and field notes were transcribed and analyzed just after they were carried out (IMV). New topics and preliminary findings were incorporated and checked in subsequent interviews. Consequently, some participants were requested to answer additional questions or provide extra information. Analyses were performed by coding transcripts and field notes and categorizing codes on the same topics in matrices. The transcripts and the matrices were reviewed by a second researcher (MMH), and codes and concepts identified from the qualitative data were agreed upon.

Structured data were organized in a case study database in Microsoft Excel (Microsoft Corporation, Inc., Redmond, WA, USA) [39]. Data were displayed in matrices, following the measures and criteria for implementation, influencing factors, and program adherence. An overview of the results was established per case (n = 5) and within each case (n = 12), and by phase (pre- and postimplementation). Cross-case synthesis was used as analytic technique to analyze the data from the multiple cases [39]. Each individual case study was treated as a separate study, and findings were aggregated. Complementary analysis of individual cases was used to identify patterns and explanations. Data analyses comprised an iterative process of identifying convergent evidence within each case and then examining and comparing the evidence across multiple case studies to draw conclusions from the study as a whole [39]. The following principles were used to report influencing factors:

– Influencing factors should be apparent in at least three of the five cases (and were regarded as even stronger if more than one respondent per case identified the same factor).

– This also meant that a factor was counted when it was a barrier in one case and a facilitator in the other.

– Influencing factors were divided by type of setting (homecare or outpatient clinic) and by phase (pre- or postimplementation) to be able to indicate patterns.

– Finally, we listed explanations with respect to influencing factors; for example, we looked at all possible reasons why patient recruitment was problematic in some cases and not in others.

Method triangulation was used to enhance the credibility of the conclusions drawn from the data and was applied as follows:

– The results of the monitor instrument (how many patients enrolled in the program) and the evaluation forms were discussed with respondents in qualitative interviews.

– Observations and field notes were checked in qualitative interviews.

– We compared the results from the barriers and facilitator questionnaire with the findings from interviews afterwards, as respondents filled in the questionnaire after the qualitative interview.

## Results

### Data collection

Table 1 presents an overview of the data collection. On two measures, data collection did not completely go as planned. In just three out of five cases, a focus group was organized. Since there was already collaboration between homecare and outpatient clinic in two cases, it was decided to do only semistructured interviews in these cases instead. Furthermore, data collection on patient recruitment appeared to be problematic in all settings. It was not possible for nurses to extract the number of eligible patients without hand-searching files, which was too time consuming. Consequently, only the number of participating patients could be retrieved.

### Description of the cases

Within the five cases, nine different healthcare organizations participated (Table 2). In two cases, the program was only delivered at the outpatient clinic for dermatology (cases 3 and 5). In another case, the program was delivered in homecare, while the outpatient clinic would refer patients to the program (case 1). Finally, in two cases, the outpatient clinic as well as the homecare organization delivered the program to their patients (cases 2 and 4).

Twelve nurses (all women) were responsible for delivering the program. Characteristics are presented in Table 2.

The way leg ulcer care was organized before implementation of the program varied. In some cases, a regional treatment protocol resulted in agreements about the care trajectory between the general practitioner (GP), homecare, and outpatient clinic (cases 3 and 5). In other cases, this was not organized, and every subsequent step in the care process was determined depending on the situation (cases 1, 2, and 4). In addition to this, the role of the nurse varied between cases. Wound care at the outpatient clinic was nurse-led in cases 3, 4, and 5, meaning that a dermatology nurse had her own clinic hours for wound care and compression therapy. A dermatologist supervised the care and usually entered the consulting room during a consult. In other outpatient clinics (case 2), patients were seen by dermatologists, and nurses entered the consulting room during a consult to execute the treatment that the dermatologist advised. Most homecare nurses were responsible for a particular district (cases 1, 2, and 4), except for the nurse practitioner in homecare who could be consulted by all districts with respect to wound care (case 1).

The number of participating patients varied between cases (Table 1). A total of 53 patients enrolled in the program, and 45 patients completed the program within the evaluation period. From the patients that have completed the program, 44% were men. The median age was 73 years, ranging from 35 to 98 years (SD = 14).

### Potential influencing factors for change

In Table 3, we list the influencing factors identified from our pre-implementation inventory. Factors that were expected to *facilitate* implementation were mainly related to the program itself and to the opportunities that the program may bring about. Eight expected *hindering* factors were identified in the pre-implementation phase.

**Table 3:** **Influencing factors identified pre**- **and postimplementation**

	**Pre**	**Post**	**Illustration**/**citation**/**score** (**data source**)
**Facilitating factor**			
Participant responsiveness: participants (nurses and patients) are positive about the content and effects of Lively Legs	X	X	- “*I think the main value of the program is that it offers a structured method you can use in your clinic hours*. *All topics that you normally would or would not discuss*, *they now will be addressed for sure with the program*.” (case 5, nurse practitioner)
			- Patients valued the program as an 8.4 on a scale from 1 to 10 on average (range 7–10) (data from patient questionnaires)
			- Nurses gave an overall program score of 3.8 on a 1 to 5 scale on average (range 3–4) (data from nurse questionnaires); nurses gave an average score of 3.6 on a scale from 1 to 5 on how satisfied they were with the extent to which they were satisfied with the achieved behavior change per patient (range 2–5) (data from evaluation forms)
Possibility to educate oneself on lifestyle counseling was appreciated by nurses	X	X	- “*I notice that I use what I learned in the Lively Legs education*… *and that I use it also in other patients*. *Personally* … *I am more aware of interview techniques*, *how to start a conversation about it*, *and showing exercises that people can use*.” (case 1, homecare nurse)
			- “*I was very enthusiastic after the educational meetings*. *I use what I*’*ve learned*, *actually in different situations*. *When I see patients at their homes now*… *you always mention lifestyle issues*… *but now I do this more completely and I say things in a different way*.” (case 2, homecare nurse)
The program gives opportunities to improve professional relationships and collaboration in the region	X		“*I hope we can improve the care for this patient group in this region*, *to come closer to some kind of collaborative care model or protocol*. *Then this project would really be regarded as successful*” (case 2, homecare manager)
Standardized care process and collaboration between homecare and outpatient clinic		X	- “*We have set up*… *how do you call it*… *a safety net*. *We just have to ask these fixed questions to every patient*…. *That it is so well organized at our clinic*, *with this standardized care process*… *I think at our clinic patients don*’*t slip through*.” (case 3, dermatology nurse)
			- “*Actually*, *I think at our clinic*, *I know every patient who comes for wound care or compression therapy*. *I have made myself an overview of which patients I have asked and which ones I have included in the program*.” (case 4, dermatology nurse)
Nurses’ own practice hours		X	- “… *We have the flexibility to schedule*… *ehm*…*You see*, *last time we could easily decide that*, *because one patient did not show up and I thought* ‘*let*’*s just do the lifestyle assessment right away*.’ *Otherwise*, *like today*, *I would schedule the appointment for next week*.” (case 5, nurse practitioner)
**Hindering factor**			
No insight in how to recruit patients for the program	X	X	- “*I just want to get those referrals*… *automatically*. *I will not*, *every time*, *because it takes me at least one hour*, *need to screen the electronic registration system*. *That just does not work*.” (case 2, specialized wound care nurse)
			- “*I don*’*t have an overview on all referrals*… *Now and then I see a form that has been filled in and then I catch that one*.” (case 2, dermatology nurse)
			- “…*Especially the problem how do you get others to refer to you*? *And how can you make sure that those agreements are guaranteed*? *When you work at the outpatient clinic you*, *are closer and you see these patients yourself*.” (case 1, nurse practitioner)
Competition between healthcare organizations	X	X	- “*I have bad luck*… *There are two main primary care practices in this village*. *Both are now only doing business with the other homecare agency*. *So*, *both will not refer patients to us*.” (case 1, homecare nurse)
			- “*I could have referred more patients to her*. *But*… *I was told not to*. *They say*, *no*, *you cannot do that because our company delivers homecare to these patients and we don*’*t want company X going to these patients as well*. *It is just fear that patients will like the nurses from company X better and that we will lose clients to our rival*.” (case 2, specialized wound care nurse)
The program is perceived as an extra task with no extra reimbursement	X		- “*The counseling should be part of the current reimbursement for venous leg ulcers*. *It would be right if the extra costs are compensated by the healthcare insurer*. *Are any finances available for this program*?” (case 3, manager outpatient clinic)
In rural regions, homecare nurses would have to drive long distances or many homecare nurses would have to be trained to cover the area	X		- “*If you train just one or two nurses for this whole region*… *You see for us it isn*’*t efficient to let them drive from village to village*, *from patient to patient*.” (case 2, homecare manager)
Who should lead the project (due to changing managers)	X	X	- Pre-implementation: in four out of five cases, the outpatient clinic manager changed positions
			- Researcher: “*So*, *if I understand you right*, *you say that part of the implementation problems can be put down to the management*?” Nurse: “*Yes*, *they don*’*t exactly accelerate the project if you know what I mean*. *It takes a long time*. *They have other kinds of problems*, *I understand that*. *But now this project may get stranded*. …*Honestly*, *I would love to go to the manager and say*, *hey there*, *you*’*ve promised this to us*.” (case 3, dermatology nurse)
Nurses’ motivation		X	- “*I should have made more inquiries before I started this project*. *What is it exactly*, *what do I have to do and what are others supposed to do*? *It*’*s just what I said before*: *I*’*ve been saddled with this project*.” (case 2, homecare nurse)
			- “*Yeah*… *well real barriers*… *no not really*… *That it didn*’*t succeed was mainly because of me*.” (case 4, homecare nurse)
Organizational preconditions (nursing time, consulting room)	X	X	- In three cases, agreements on nursing time were not (totally) met. There were no problems with respect to the availability of consulting rooms. Nurses valued the extent to which organizational preconditions were met with 3.3 on a scale from 1 to 5 on average (data from nurse questionnaires).
			- “*I can hardly schedule appointments*. *Because*, *well I only work on Wednesdays*. *I want to combine the counseling with wound care consultations and*… *well if the doctor is not doing clinic hours that Wednesday for example*… *It is difficult to arrange and before you know it is three weeks later*.” (case 2, dermatology nurse)
Knowledge and support of colleagues		X	- Colleagues scored a 4.5 on a scale from 1 to 5 on average (range 4.2–5.0) with respect to knowledge of the program (data from questionnaire colleagues)
			- Nurses scored a 3.3 on a scale from 1 to 5 on average (range 2–5) to the extent to which colleagues were supportive of them in implementing the program (data from questionnaire nurses)

In almost all cases, participants stated that it is not clear what would be the best place to recruit as many patients for the program as possible. Moreover, they said that patients could not be easily grouped and identified from patient information systems due to different diagnostic and procedure codes (*e*.*g*., in homecare, venous leg ulcer patients are coded as either “wound care” or “compression therapy”). Furthermore, competition between healthcare organizations was identified as a potential barrier. Nurses and dermatologists stated that due to different financial incentives, organizations might have reservations about referring patients to the lifestyle program. Connected to this, reimbursement was mentioned as a barrier mainly by managers and dermatologists. Subsequently, some practical barriers were identified, for instance, how many nurses should be trained to cover rural areas, whether nurses have sufficient time to deliver the program, and how to deal with changing clinic managers during the implementation period at outpatient clinics.

### Implementation strategies

After this, implementation strategies were selected. These implementation strategies and the extent to which they were carried out can be found in Table 4. Training of the nurses, managerial agreements on nursing time, and monitoring the number of participating patients were regarded as elementary to program implementation and therefore compulsory. However, each setting could use some of the other implementation strategies as desired, depending on the wishes and needs of particular settings. Next to this, some strategies were carried out by researchers: a practice visit to discuss possible problems and the development of a website with information and program materials.

**Table 4:** Delivery of implementation strategies

	**Case 1**	**Case 2**	**Case 3**	**Case 4**	**Case 5**	**Cases** (**n** = **5**)	**Settings** (**n** = **12**)
Agreements on (extra) nursing time^a^	Yes	Partly	Yes	Partly	No	4/5	8/12
Training Lively Legs (two days)^a^	Yes	Yes	Yes	Yes	Yes	5/5	12/12
Training Lively Legs after implementation (Â½ day)^a^	Yes	Yes	Yes	Partly	No	4/5	10/12
Monitor patient recruitment^a^	Yes	Yes	No	Yes	Yes	4/5	10/12
Monthly feedback on patient recruitment^b^	Yes	Yes	No	Yes	Yes	4/5	10/12
One practice visit by researchers^b^	Yes	Yes	Yes	Yes	Yes	5/5	11/12
Website with information and program materials^b^	Yes	Yes	Yes	Yes	Yes	5/5	12/12
Communicate the referral procedure in team^c^	Yes	Yes	Yes	Yes	Yes	5/5	12/12
Determine referral procedure with external referrers^c^	Yes	Partly	No	Partly	No	3/5	4/12
Sending a letter to external referrers^c^	No	No	No	Yes	No	1/5	1/12
Forum on website for questions and feedback^c^	No	No	No	Yes	Yes	2/5	2/12
Ask researchers for support^c^	Yes	Yes	Yes	Partly	Yes	5/5	6/12
Hand out information leaflet to referrers^c^	Yes	Partly	No	Yes	No	3/5	5/12
Publish information on company website/paper^c^	No	Yes	No	Yes	Yes	3/5	7/12
Inform others about lifestyle in leg ulcer patients^c^	Yes	No	No	No	No	1/5	1/12
Hand out cards with lifestyle advices and referral procedure (only in cases 4 and 5)^c^	-	-	-	Yes	No	1/2	2/5
Number of strategies carried out of planned (range)^d^	12/15	12/15	7/15	15/16	9/16		
	(11–12)	(8–12)	(7)	(4–14)	(9)		

^a^Compulsory implementation strategies. ^b^Implementation strategies carried out by researchers. ^c^Implementation strategies to be used as desired. ^d^Presents the range between the healthcare settings within the case.

Training of nurses, as a first strategy, consisted of four elements: knowledge about health behaviors in patients with venous leg ulcers, knowledge about behavior change, training on motivational interviewing skills, and training on program implementation. Nurses were trained by a group of trainers consisting of a nurse scientist, a clinical psychologist/cognitive behavioral therapist, and a professional trainer in motivational interviewing. With regard to patient recruitment, a screening instrument was developed. Additionally, nurses were asked to monitor weekly the referrals to the Lively Legs program using an online monitor tool. They received feedback per setting on a monthly basis. The rationale behind this was to get insight in the number of potential patients and where the majority was treated. A number of strategies were developed to inform referrers and colleagues. These were to be used as desired: a website (with information, program materials, and an online forum), a text to be used to inform patients about the lifestyle program, and educational materials to inform colleagues and referrers about Lively Legs.

### Evaluation

The Lively Legs program was carried out with a moderate to good adherence to protocol (65%–90%). Detailed results on program adherence are described in Additional file 1. Main elements of the program were generally delivered as planned. There was some variation in goal setting and the extent to which patients’ motivation and self-efficacy were assessed. Also, barriers for behavior change were not always explored by some nurses. Our analyses, however, focused on influencing factors for implementation. First, the factors that influenced implementation will be reported, including the results on the quality of the delivery of implementation strategies. This is followed by the results on perceived implementation success.

Various factors influenced the implementation of the Lively Legs program. These—and their illustrations—can be found in the postimplementation column in Table 3. Reaching and recruiting patients was regarded as the most difficult part of the program to implement. This factor was also mentioned most pre-implementation. When comparing the settings, patient recruitment was higher at the outpatient clinic than in homecare. In three cases (cases 3, 4, and 5), all at outpatient clinics, nurses declared that all patients with venous leg ulcers were asked to participate in the program. These nurses had their own practice hours, and this was sometimes in combination with a standardized care process for this patient group, where a designated step in the treatment protocol was created to define when to recruit patients and who should do this.

Participants who reported that patient recruitment was problematic felt they were dependent on GPs or dermatologists for referrals: “Maybe if you put in more time…a lot more time. Maybe, you should just keep telephoning those GPs. (…) I think, if you can just lean on the support of GPs… Well I think that’s the biggest issue” (case 4, homecare nurse). In addition to this, nurses in homecare and those working at an outpatient clinic without a standardized care process explained that they had no overview of eligible patients themselves. Apart from that, homecare nurses reported a low concentration of venous leg ulcer patients in their particular district, indicating that there were not enough eligible patients: “Implementation is doable in theory, but I wonder if it’s sensible, at least in homecare. If I see the limited numbers of patients we are able to recruit in homecare, I wonder: wouldn’t it be better to do this elsewhere?” (case 2, homecare nurse). Connected to this, some participants stated that the program should rather or should also be focused on patients with venous insufficiency who do not (yet) have a wound. A dermatology nurse said, “I think from the viewpoint of prevention… the program should also be offered in homecare, because the chronic venous insufficiency problems may linger in homecare. At the outpatient clinic, we don’t see these patients that often. We see them when it’s too late” (case 3, dermatology nurse).

With respect to patient recruitment, patients were specifically asked if they wanted to participate in the Lively Legs program. Nurses said that intrinsically motivated patients participated in the program and that they sometimes had difficulties in motivating other patients. “Most of them are older than 70 and sometimes even older than 80. They are hesitant to join yet another thing. Or they are afraid it will cost them money. Sometimes they only agree to join if it can be combined with their other appointments at the clinic” (case 2, specialized wound care nurse).

From interviews it appeared that working hours of participating nurses was a factor of interest. For example, in case 2, two nurses at the outpatient clinic were working for less than 12 hours per week. Managers and nurses explained in interviews that this resulted in difficulties in coordinating the program and planning counseling sessions. Apart from time, nurses’ motivation was also considered as an influencing factor. Some of them stated that, as a nurse, it was important to be dedicated to giving lifestyle counseling. In cases 2 and 4, nurses had not specifically chosen to deliver the program. In these settings, patient recruitment rates were lower, as well as nurses’ satisfaction with the program.

Setting seemed an influencing factor with respect to the content of the counseling. This was identified in interviews and in field notes of observed counseling sessions. Patients at the outpatient clinic received practical tips of a different kind than in homecare, for instance, on how to take off compression stockings in an easy way or what kind of cream to use for skin care. Also, referrals to bandage therapists were made more easily at the outpatient clinic than in homecare. In one case, a homecare nurse discovered that one of the patients did not have compression therapy or stockings: “I don’t dare to say that she should have had them. Because, well … I don’t even know if she’s had the right diagnostics. I don’t think so” (case 1, nurse practitioner).

From structured data, several factors were checked to see if and how they were of importance to the implementation of Lively Legs. First, the extent to which strategies were carried out as planned varied (see Table 4), ranging from 4 to 14 strategies. Training of the nurses was the strategy that was carried out in all settings and was judged as most useful for implementation. Although participants stated in the preparation phase that informing and educating colleagues and referrers was important, the majority of the nurses did not do this. “I now notice, I am occupied with other things again. Then, it’s just what I said… It’s also a bit my own fault that I don’t give it all it takes” (case 1, nurse practitioner).

Participants reported that they were satisfied with the range of strategies. “If you have patients who participate, and if you have completed the training and have the program materials, …I think then you can just implement this” (case 4, dermatology nurse). Training and agreements on nursing time were regarded as the most facilitating strategies. According to nurses, monitoring and feedback on patient recruitment was not a helpful strategy to implementing the program because the feedback information was of no extra value to them.

Patients valued the program with a mean score of 8.4 (Table 3). Nurses gave an overall program score of 3.8 on average on a scale from 1 to 5, and in interviews they also underlined their satisfaction with the content of the program.

With respect to perceived implementation success, nurses scored this ranging from 4.9 to 7.0 on a scale from 1 to 10 (Table 5). However, almost all nurses expected that implementation would be better in a half-year’s time. As elaborated on earlier, patient recruitment varied between cases, and in three cases, the standard was met of recruiting one patient per month for the lifestyle counseling program.

**Table 5:** Implementation success

**Criteria**		**Case 1**	**Case 2**	**Case 3**	**Case 4**	**Case 5**	**Data source**
Perceived implementation success	Implementation score (1–10)	5.0	4.9	6.0	5.3^a^	7.0	Interviews with nurses
	Half-year implementation expectation (1–10)	5.0	5.4^a^	8.5	7.5	8.0	Interviews with nurses
Patient recruitment	Patients enrolled in program (n = 53)	10	18	14	6	5	Monitor
	Patients completed program within evaluation (n = 45)	10	18	7	5	5	Monitor and nurses’ registration
	One new patient per month	No	No	Yes	Partly^a^	Yes	Monitor
	All eligible patients are recruited according to nurse	No	No	Yes	Partly*	Yes	Interviews with nurses

^a^There was variation between the healthcare settings within the case.

### Linking influencing factors to perceived implementation success

From the hindering factors, four were referred to before and after implementation. These were problems related to patient recruitment, competition between healthcare organizations, insufficient nursing time, and lack of support of management. The factors related to patient recruitment were mentioned most and as most hindering for program implementation. Conversely, the most facilitating factors for implementation were also connected to patient recruitment. These were the existence of a standardized care process and nurses’ own practice hours and possibilities to overview patient recruitment.

## Discussion

### Main findings

The aim of this study was to identify which factors hindered and facilitated the implementation of Lively Legs in outpatient clinics for dermatology and in homecare. Factors were mainly found at the organizational level. The most difficult part of the program to implement was the organization of reaching and recruiting patients. In this study, we found that if a nurse coordinated a standardized care process with a clear treatment protocol to tie Lively Legs into, this resulted in better patient recruitment and better implementation. Other factors that facilitated implementation were sufficient nursing time and motivated nurses to deliver the program.

Implementing the Lively Legs program at the outpatient clinic for dermatology had a number of advantages compared to implementation in homecare organizations. It took less time to coordinate recruitment, possibly due to a higher concentration of patients at outpatient clinics and less dependency on others for referrals. Also, barriers related to market competition were avoided. Furthermore, the outpatient clinic showed salience in giving practical advice and tips to patients. Implementation in homecare is only possible when ulcer care is well organized and when sufficient nurses are trained to counsel patients. The need for a clear treatment protocol and collaboration between outpatient clinic, homecare, and GPs emerged to be important in every case in this study.

The fact that patient recruitment was difficult in some cases and that there was a prominent need for a clear treatment protocol to ensure the continuity of care in every case in this study revealed more general issues with respect to leg ulcer care. Leg ulcer care was fragmented in some cases. At those settings, it was difficult to organize patient recruitment and thus implement the Lively Legs program. Moreover, this fragmented care delivery revealed that quality improvement is needed, in particular with respect to the handover of care between GP, outpatient clinic, and homecare.

A case study design was used to investigate the implementation within its real-life context and to explore influencing factors before, during, and after implementation. It appeared that participants were able to identify relevant influencing factors beforehand and that these were mostly confirmed during and after implementation. However, reimbursement was regarded as an influencing factor beforehand but was not mentioned afterwards, indicating that this factor seemed only of influence in the decision-making process. Furthermore, the context and setting purposefully varied between cases, and this appeared to be crucial to understanding the influencing factors for implementation, in particular, the influence of the organization of leg ulcer care; the extent to which collaboration between homecare, outpatient clinic, and GP was established; and the extent to which the nurse coordinated the care process. By closely looking at what happened, it was made apparent that a clear treatment protocol and continuity of care facilitates the implementation of Lively Legs. Conversely, a lack of well-organized care does not only hinder implementation of the lifestyle program but also raises questions about the quality of care for this patient group.

Although similar initiatives to promote better adherence to leg ulcer treatment were reported in other countries, no implementation studies have been performed yet. Nevertheless, several factors that influenced the implementation of Lively Legs have also been reported in some studies in other patient groups. For example, Lorig *et al*. (2005) have pointed out that program reach and lack of referrals from physicians is often problematic with respect to implementing lifestyle interventions [34]. Direct outreach and communication with patients proved to be the most effective means of recruitment. This is in line with our findings that nurses should overview the recruitment of patients themselves. Furthermore, in our study, nurses said that only motivated patients participated in the program. Similar recruitment bias often occurs in lifestyle interventions [44].

Program adherence was moderate to good, implying that the program can be adequately carried out in daily practice. Variation between cases was found with respect to goal setting, assessing patients’ motivation and self-efficacy, and exploring patients’ barriers and facilitators for behavior change. This indicates that motivational interviewing skills need continuing attention, for example, through repetitive training or coaching on the job. This finding is in line with Whittemore *et al*. (2009) and Griffin *et al*. (2009), who reported that motivational interviewing was the most challenging aspect of their protocol to implement and that continued training was needed [35,45].

### Strengths and limitations

The strength of using a case study design lies in the opportunity to study the implementation process in real-life settings and collect multiple types of data, enabling development of an in-depth picture [39]. By selecting cases that varied in the way cooperation took place between homecare and outpatient clinic, we were able to obtain a broad picture of everyday practice. The aim of this study was not to count how many times implementation succeeded in a controlled situation or to assess the effectiveness of certain implementation strategies, but to understand if and how program implementation was possible. Carrying out such an implementation study indicates that external researchers can only partly influence an implementation process from the outside; for instance, there is no guarantee that participants use implementation strategies as intended. And even if they do, contextual factors can still play an important role. This makes research complex and unruly. But, by closely following a relatively small number of implementation trajectories, we succeeded in identifying valuable factors that influenced the implementation. In addition to this, this study provided an opportunity to gain more understanding of the way leg ulcer care is organized.

However, it may be that some nurses in this study had insufficient insight in events or influencing factors at the management level or regional level to provide valid answers. But, the variation in answers and gained insight in implementation difficulties do not point in the direction of possible unreliable answers. Furthermore, due to low patient recruitment, some nurses may not have been able to master their skills in program delivery. Program adherence could have been affected by this. Another critical reflection concerns the fact that evaluation took place after seven months in cases 1, 2, and 3 and after four months in cases 4 and 5. Due to time constraints, it was not possible to keep both evaluation periods the same. There might be a possibility that other results would have been obtained after a longer period before evaluation, but our results from all cases point in the same direction, indicating that the findings on our main question are trustworthy. Furthermore, we would like to comment that in this study, the selected implementation strategies were mostly voluntary and on the micro-level. In hindsight, it might have been necessary to first make structural changes to the care process before implementing the program or to include structural changes as one of the implementation strategies. On the other hand, these structural changes would not have been feasible within the time and scope of this research project.

In this study, we used the framework of Hasson (2010) to guide us in collecting and organizing the data. For this purpose this sufficed very well. On the one hand, the framework gives structure; on the other hand, it is still generic enough to explore how factors exactly influenced the implementation of this explicit lifestyle program. At the same time, we acknowledge that other frameworks such as these are available and could have been equally suitable.

### Implications

With respect to leg ulcer treatment, further research is needed on effects of process redesign to improve the continuity and quality of care in such a way that prevention is also included. Next to this, expanding lifestyle counseling to patients with chronic venous insufficiency, as an early stage of leg ulceration, should also be investigated.

## Conclusion

In summary, we conclude that the main influencing factors for implementing Lively Legs are at the organizational level. The organization of patient recruitment was most difficult. When lifestyle counseling is integrated in regular care as part of a standardized treatment protocol, it helps to guarantee that all patients can at least take part in the first step of the program. Other factors that facilitated the implementation were selecting dedicated nurses for delivering the program and facilitating them with sufficient nursing time and tools to coordinate the care process. We found that nurses were able to deliver the program adequately, in such a way that patients as well as nurses were very satisfied.

This study suggested that the Lively Legs program can best be implemented at the outpatient clinic for dermatology. The specific influencing factors may be relevant more widely in programs for implementing lifestyle interventions in different healthcare settings. The value of using a case study design is that an in-depth picture is obtained of how and why implementation is possible in different settings. Furthermore, it also shed light on a more general issue, that is, that leg ulcer care was often fragmented, indicating that quality improvement is needed in particular with respect to treatment protocols and the handover of care between GP, outpatient clinic, and homecare.

## Competing interests

MW is Editor in Chief of Implementation Science. All decisions on this paper were made by another editor.

## Authors' contributions

IMV, MMH, and TV contributed to study design. AWE and MW provided advice on study design. IMV and MMH performed the data collection and analysis. IMV was responsible for manuscript preparation. MMH, AWE, MW, and TV provided critical comments on the manuscript. All authors read and approved the final manuscript.

## Supplementary Material

Additional file 1 Program adherence Lively Legs. Click here for file
