# Supplementary material for: Factors influencing the implementation of a lifestyle counseling program in patients with venous leg ulcers: a multiple case study
Source: Implement Sci. 2012 Oct 26;7:104. doi: 10.1186/1748-5908-7-104 (PMC3520793; doi:10.1186/1748-5908-7-104)
Supplement: Additional file 1 — Program adherence Lively Legs. [file 1748-5908-7-104-S1.docx]

**Additional data file 1 Program adherence Lively Legs**

Program adherence is the extent to which the program is delivered as intended, and was measured by assessing frequency, duration and content of counseling sessions. Based on this assessment a coverage score was computed.

- *Frequency and duration* of counseling sessions were registered by nurses on an evaluation form.
- The *content* of the counseling was categorized assessed by analyzing patient files using several measures and by interviewing nurses who conducted the counseling sessions.
  - Conducting lifestyle anamneses: patient files were reviewed to determine if the anamneses were carried out (yes/no).
  - Handing out information leaflets: nurses were asked to how many patients the venous leg ulcer information leaflet was handed out.
  - Goal setting: patient files were analyzed if there were any formulated goals for behavior change formulated and written down. Each patient should have formulated at least one goal.
  - Motivation assessment: patient files were analyzed if patients’ motivation for behavior change was assessed and registered (yes/no). Motivation for change should have been assessed at least one time during counseling.
  - Self efficacy assessment: patient files were analyzed if patients’ self efficacy for behavior change was assessed and registered (yes/no). Patients’ self efficacy should have been assessed at least one time during counseling.
  - Barriers and facilitators assessment: patient files were analyzed to assess if barriers and facilitators for behavior change were discussed and written down (yes/no). This should have been assessed at least one time during counseling.
- *Coverage* was defined as the percentage of program elements delivered as intended. A coverage score was computed by dividing the number of elements conducted in counseling per patient by the number of total elements per patient that should have been conducted.

For this study, a coverage score of 80% to 100% was regarded as high program adherence; a coverage score between 50% - 80% was regarded as moderate, and 50% or less as low program adherence.

Table 1 presents the results on program adherence. There was variation in frequency and duration of the program between cases. Both were less than designed. An explanation for the variation is that in cases 2, 4 and 5 most counseling sessions were planned right after wound care. In cases 1 and 3 an appointment was especially made for the counseling, possibly with less time-pressure. There was also some variation in goal setting and assessing patients’ motivation, self efficacy or barriers for behavior change. From the patient files it came clear that in 10 of the 38 files no behavioral goals were formulated and written down. In some files, nurses had written down a remark: that the patient did not wanted to change at all, wanted to take some time to think about an appropriate goal, or that it was not necessary because the patient already promised to follow the advices. Even though a goal was formulated, patients were not always asked about their motivation or self efficacy to change their behavior. Why this was not always carried out by nurses, could not be retrieved from analysis of the patient files.

Table 1 Program adherence Lively Legs

| **Program adherence** | **Criteria** | **Case 1** | **Case 2** | **Case 3** | **Case 4** | **Case 5** | **Data source** |
| --- | --- | --- | --- | --- | --- | --- | --- |
| Frequency | Mean number of consultations (range) | 100%  2.7  (1-4) | 100%  2.5  (1-4) | 100%  2.3  (1-3) | 100%  2.2  (1-3) | 100%  1.4  (1-2) | Evaluation forms filled in by nurses (n=43) |
| Duration | Mean in minutes (range) | 100%  159  (60-260) | 100%  108  (45-175) | 100%  124  (90-180) | 100%  98  (90-120) | 100%  60  (50-75) | Evaluation forms filled in by nurses (n=43) |
| Content | Anamneses | 100% | 100% | 100% | 100% | 100% | Patient files (n=38)*** |
|  | Information leaflet | 80% | 100% | 100% | 100% | 100% | Interviews with nurses (n=12) |
|  | Goal setting | 57% | 95% | 71% | 38%* | 60% | Patient files (n=38) |
|  | Motivation assessment | 43% | 83% | 86% | 25%* | 40% | Patient files (n=38) |
|  | Self efficacy assessment | 29% | 83% | 86% | 25%* | 40% | Patient files  (n=38) |
|  | Barriers and facilitators assessment | 14% | 58% | 86% | 38%* | 40% | Patient files  (n=38) |
| Coverage** | % elements delivered per patient | 65% | 90% | 91% | 66%* | 73% |  |

* There was variation between the healthcare settings within the case.

** Coverage = elements carried out per patient divided by total elements that should have been carried out per patient. The elements were: frequency, duration, anamneses, information leaflet, goal setting, motivation assessment, self efficacy assessment, barriers and facilitators assessment.

*** Five patient files were missing, despite repeatedly asking nurses to send these files.
